# Thymoquinone restores liver fibrosis and improves oxidative stress status in a lipopolysaccharide-induced inflammation model in rats

**Published:** 2017

**Authors:** Fereshteh Asgharzadeh, Rahimeh Bargi, Farimah Beheshti, Mahmoud Hosseini, Mehdi Farzadnia, Majid Khazaei

**Affiliations:** 1 *Department of Physiology, School of Medicine, Mashhad University of Medical Sciences, Mashhad, Iran*; 2 *Neurocognitive Research Center, School of Medicine, Mashhad University of Medical Sciences, Mashhad, Iran*; 3 *Departments of Pathology, School of Medicine, Mashhad University of Medical Sciences, Mashhad, Iran*; 4 *Neurogenic Inflammation Research Center, Department of Physiology, School of Medicine, Mashhad University of Medical Sciences, Mashhad, Iran*

**Keywords:** Inflammation, Thymoquinone, Liver, Fibrosis

## Abstract

**Objective:**

Liver fibrosis is the primary sign of chronic liver injury induced by various causes. Thymoquinone (TQ) is the major ingredient of *Nigella sativa* with several beneficial effects on the body. In the present study, we aimed to investigate the effect of TQ on liver fibrosis in a lipopolysaccharide (LPS)-induced inflammation in male rats.

**Materials and methods:**

Fifty male Wistar rats were randomly divided into five groups (n=10 in each group) as follow: (1) control; (2) LPS (1 mg/kg/day; i.p); (3) LPS+TQ 2 mg/kg/day (i.p) (LPs+TQ2); (4) LPS+TQ 5 mg/kg/day (LPS+TQ5); (5) LPS+ TQ 10 mg/kg/day (LPS+ TQ10). After three weeks, blood samples were taken for evaluation of liver function tests. Then, the livers were harvested for histological evaluation of fibrosis and collagen content and measurement of oxidative stress markers including malondialdehyde (MDA), total thiol groups, superoxide dismutase (SOD) and catalase activity in tissue homogenates.

**Results:**

LPS group showed higher levels of fibrosis and collagen content stained by Masson’s trichrome in liver tissue with impaired liver function test and increased oxidative stress markers (p<0.05). Treatment by TQ restored liver fibrosis, improved liver function tests and increased the levels of anti-oxidative enzymes (SOD and catalase), while reduced MDA concentration (p<0.05).

**Conclusion:**

Treatment by TQ restores inflammation-induced liver fibrosis possibly through affecting oxidative stress status. It seems that administration of TQ can be considered as a part of liver fibrosis management.

## Introduction

Liver fibrosis is the primary sign of all types of chronic liver injury induced by a variety of factors such as chronic hepatitis, alcohol abuse, or nonalcoholic steatohepatitis (NASH) (Bataller and Brenner, 2005 ▶). The prevalence of liver fibrosis is around 2.8% (Poynard et al., 2010 ▶). Progressive fibrosis has been implicated in cirrhosis and eventually death due to liver failure (Desmet et al., 1994 ▶). Liver fibrosis results from intemperate collection of extracellular matrix (ECM) proteins including type I collagen following liver injury (Friedman, 2000 ▶). Liver fibrosis results in cirrhosis, liver failure, portal hypertension and hepatocellular carcinoma which is the third cause of cancer-related deaths worldwide (Liver, 2012 ▶; Tsochatzis et al., 2014 ▶). Although genomic factors are associated with liver fibrosis, but environmental agents such as viral or bacterial infections and malnutrition are considered as more common causes (Bataller and Brenner, 2005 ▶). Studies indicated that inflammation-induced by lipopolysaccharide (LPS) is involved in fibrosis processes (Ceccarelli et al., 2015 ▶). LPS is an endotoxin which is a cell-wall component of gram negative bacteria which binds to toll-like receptors (TLRs) such as TLR4 and leads to inflammation by promoting oxidative stress and inflammatory markers production (Schwabe et al., 2006 ▶). 

In the liver, activation of hepatic stellate cells (HSCs) and Kupffer cells result in liver fibrosis possibly through increased expression of TLR4 on them. Also, the release of free radicals and cytokines induced by LPS is responsible for activation of hepatic stellate cells (HSCs), and it seems that these cell are direct targets of LPS *in vitro* and *in vivo* (Sánchez-Valle et al., 2012 ▶). Thus, it has been suggested that TLRs connect inflammation-induced hepatic injury and fibrogenic signals (Paik et al., 2003 ▶).

Thymoquinone (TQ) is the major active compound of *Nigella sativa* with anti-inflammatory, anti-oxidant and anti-fibrotic properties (Amin and Hosseinzadeh, 2016 ▶; Bai et al., 2013 ▶). The beneficial effects of TQ have been studied on several diseases such as bronchitis, diabetes, cardiovascular diseases, rheumatism, cancer, asthma, neuronal disorders and liver injury like fibrosis (Gholamnezhad et al., 2016 ▶; Nagi et al., 2011 ▶; Woo et al., 2012 ▶). Therefore, based on these findings, the use of TQ as an anti-inflammatory and anti-oxidant chemical could be a reliable therapeutic intervention. In this study, we assessed serum levels of liver enzymes (e.g. aspartate aminotransferase (AST), alanine aminotransferase (ALT) and alkaline phosphatase (Alk-P), and oxidative stress parameters in liver tissue, and we evaluated histological parameters and liver fibrosis to find the effect of different doses of TQ on LPS-induced liver fibrosis in male rats. 

## Materials and Methods

**Animals**

Fifty male Wistar rats weighing 240±10 g were used in this study. The animals were housed in groups of 5 in each cages and kept under standard situation (temperature 22±2 °C, humidity of 54 ± 2% and 12h/12h light/dark cycle) with free access to food and water, *ad libitum*. All experimental protocols were approved by the Ethics Committee of Animal Research, Mashhad University of Medical Sciences, Mashhad, Iran. 

**Experimental protocol**

The animals were randomly divided into five groups as follow (n=10 in each group): 

Control: received saline injection, intraperitoneally (i.p) for three weeks.

LPS: received daily injection of LPS (1 mg/kg; i.p) for three weeks.

LPS+TQ 2 mg/kg (LPS+TQ 2): received LPS plus TQ 2mg/kg/day (i.p).

LPS+TQ 5 mg/kg (LPS+TQ 5): received LPS plus TQ 5 mg/kg/day (i.p).

LPS+ TQ 10 mg/kg (LPS+ TQ 10): received LPS plus TQ 10 mg/kg/day (i.p).

LPS and TQ were purchased from Sigma-Aldrich (Sigma Chemical Co, USA) and freshly dissolved in sterile saline prior to injection. After three weeks, blood samples were taken from orbital sinus and centrifuged. Next, the sera were kept at -70°C for further analysis. Then, the animals were sacrified and the middle lobe of livers were dissected and washed with saline. Then, a part of the middle lobe of livers was put in formalin 10% solution and the remaining was immediately kept at -70°C, for evaluation of tissue oxidative stress markers. 

**Evaluation of oxidative stress markers in liver tissue**

*Determination of Malondialdehyde (MDA)*

Malondialdehyde (MDA) content as an indicator of lipid peroxidation in liver tissue was determined. Liver tissue was homogenized and an aliquot of the homogenate was added to a reactive substance, thiobarbituric acid. The absorbance of the mixture was measured using spectrophotometer at 535nm against a blank and the MDA content of samples were determined using a standard curve.

*Determination of total thiol groups (SH)*

Total thiol group in liver homogenates was measured by a biochemical assay using dithionitrobenzoic acid (DTNB). Reduced glutathione was taken as the standard for plotting the standard curve. Supernatants were incubated with DTNB in 1 mL Tris-EDTA buffer (pH 8.6). Then, the mixture was incubated for 10 min at room temperature and the absorbance was measured spectrophotometrically at 412 nm. The GSH content was calculated using a standard curve.

*Determination of superoxide dismutase (SOD)*

Evaluation of SOD activity was done based on the production of superoxide dismutase through auto-oxidation of pyrogallol and inhibition of conversion of MTT to formazan. Next, DMSO was used to dissolve formazan and produce stable colors. Finally, the absorbance was read at 570 nm.

*Determination of catalase*

The activity of catalase was determined according to the rate of decomposition of hydrogen peroxide (H_2_O_2_) by catalase by spectrophotometer at 240 nm. 

**Analysis of biochemical parameters in serum**

Assessment of AST, ALT, Alk-P, albumin and total protein concentrations were measured in blood samples using commercially available diagnostic kit (Alsaif, 2007 ▶).

**Histological evaluation**

The middle lobe of livers from all animals were dissected and washed with saline, and then, fixed in 10% neutral buffered formalin for 24-72 hr. After dehydration, the tissues were embedded in paraffin, and cut into 5-μm thick sections using a microtome. The paraffin-embedded sections were stained with Hematoxylin-Eosin (H&E) and Masson’s trichrome stains. The sections were examined under light microscopy (X40 magnification). Fibrosis was measured as the percent of fibrotic tissue regions stained with Masson trichrome. The zone of fibrosis was evaluated utilizing Image J software.

**Statistical analysis**

The data were expressed as mean ± SEM. One way ANOVA test followed by Tukey as the *post hoc* test, was used for making comparison between different groups. The statistical significance was considered when p<0.05.

## Results

**Effects of TQ on oxidative parameters in liver tissue**

**Figure 1:**
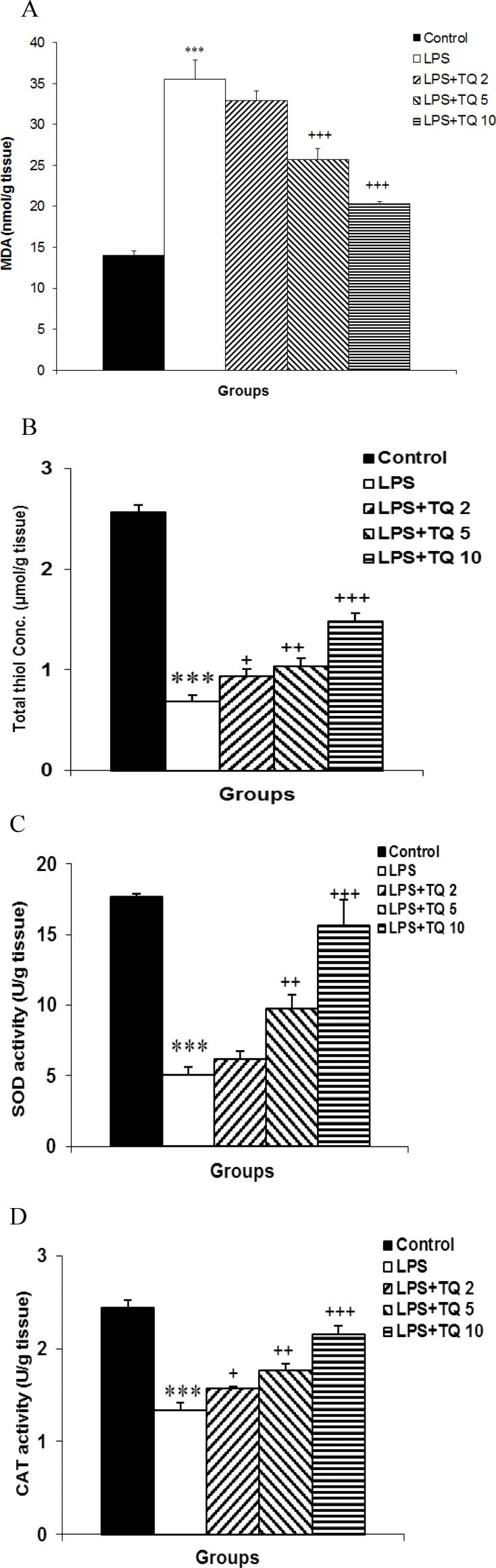
Comparison of MDA (A), total thiol (B), SOD (C) concentrations and catalase (D) activities in liver tissue among experimental groups. Data are expressed as mean ± SEM (n= 10 in each group). *** p<0.001 compared to control group; + p<0.05, ++ p<0.01 and ^+++^ p<0.001 compared to LPS group

As shown in Figure 1A, the LPS group had higher MDA concentration, as an index of lipid peroxidation, compared to control (p<0.001). TQ decreased the levels of tissue MDA as MDA levels in LPS+TQ5 and LPS+TQ10 were significantly different from those of LPS group (p<0.001). LPS+TQ2 group had lower tissue MDA concentration than LPS; however, it was not statistically significant.

Evaluation of total thiol groups, and SOD and catalase activity in liver tissue indicated that their levels were lower in LPS group than control (p<0.001) (Figure 1B-D). Administration of TQ resulted in a dose-dependent increase in total thiol groups, and SOD and catalase activity in liver issue, and these increases were statistically significant compared to LPS group (p<0.05). 

**Effects of TQ on liver function test**

**Figure 2:**
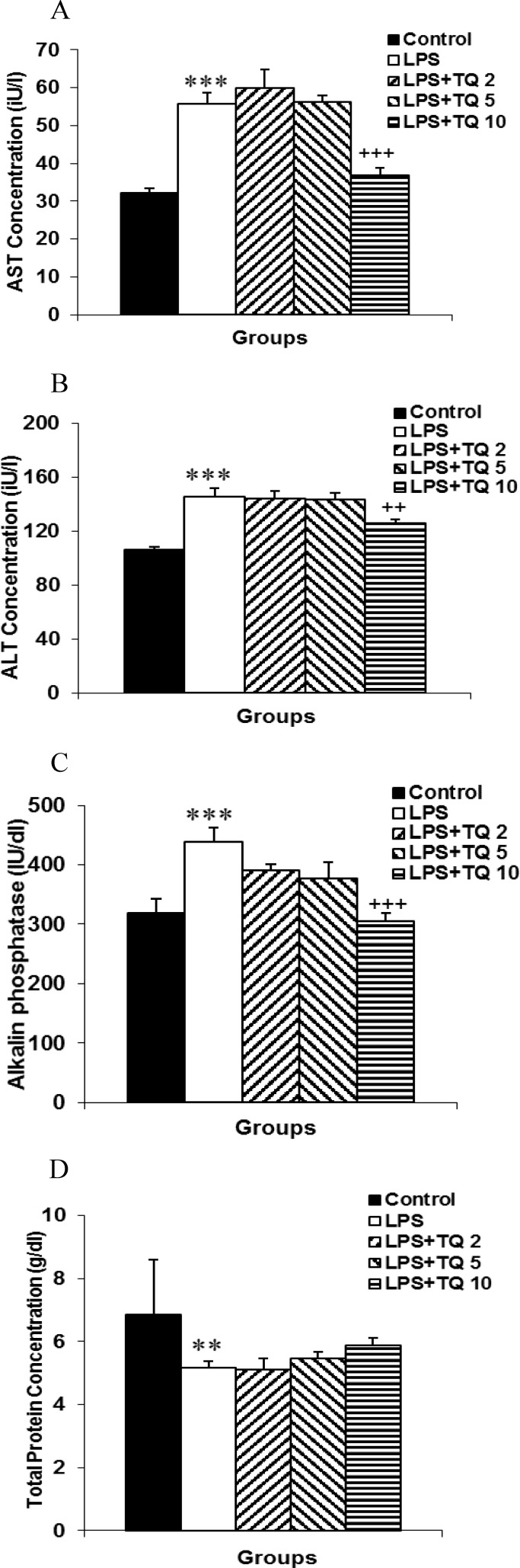
Comparison of serum AST (A), ALT (B), Alk-P (C) and total protein (D) concentrations. Data are expressed as mean ± SEM (n= 10 in each group). **p<0.01 and ***p<0.001 compared to control group. ++ p<0.01 and +++p<0.001 compared to LPS group

**Figure 3:**
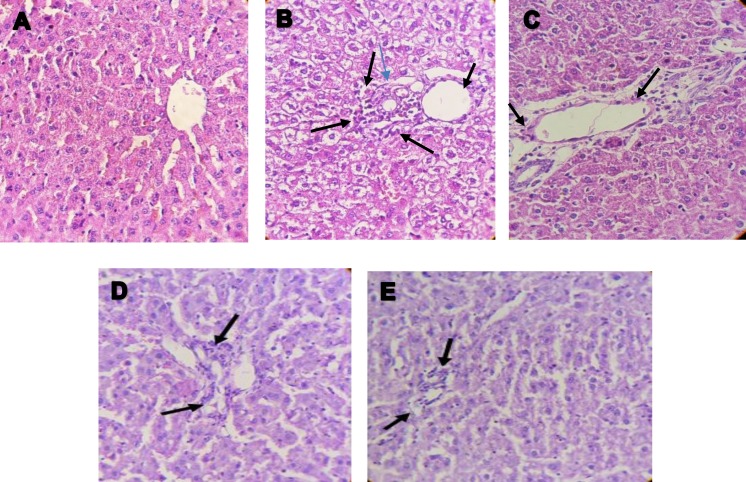
A-E show H&E-stained sections of liver tissue (X40). Arrows indicate infiltration of inflammatory cells. A: Control; B: LPS; C: LPS+TQ2; D: LPS+TQ5 and E: LPS+TQ10

AST and ALT concentrations are routinely used as indices of hepatic function. Our results showed that both ALT and AST levels significantly increased in LPS group as compared to control group (p<0.001 for both cases) (Figures 2 A and B). Administration of TQ 2 and 5 mg/kg/day could not alter serum AST and ALT concentrations. However, serum AST and ALT levels were significantly lower in LPS+TQ10 group (p<0.001 for both cases). The same results were also observed in the case of Alk-P concentration (Figure 3C). Serum total protein was at lower levels in LPS group and it was not affected following treatment with different doses of TQ (Figure 3D).

**Histological findings**

**Figure 4:**
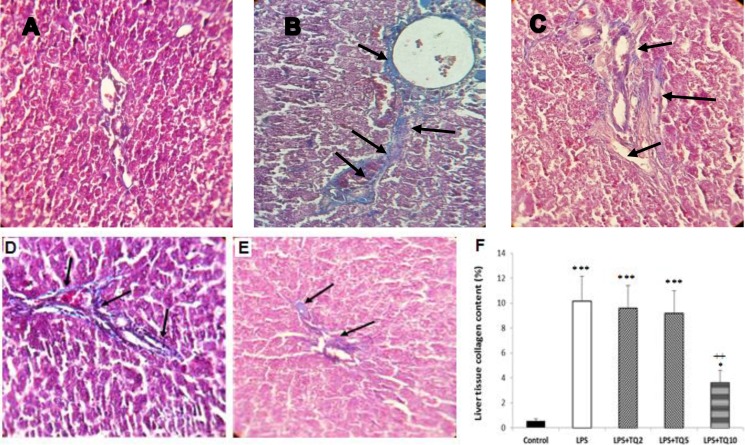
Masson's trichrome-stained sections of liver tissue demonstrating collagen deposition (blue color indicated by arrow) or fibrotic changes in control group (A), LPS group (B), TQ2+LPS group (C), TQ5+LPS group (D) and TQ10+LPS group (E). Collagen content of liver tissue was higher in LPS group and it was reduced by different doses of TQ (F). * p<0.05 and *** p<0.001, compared to control; ++ p<0.01 compared to LPS, LPS+TQ5 and LPS+TQ2

Figures 3 and 4 illustrate liver tissue samples stained with H&E and Masson's tichrome, respectively. H&E-stained images demonstrated a normal liver lobular architecture with central vein and radiating hepatic cords in control group (Figure 3A). In contrast, LPS group indicated increments in lymphocyte and neutrophil infiltration in the central and portal areas (Figure 3B). In LPS-treated animals that were treated with TQ 2, 5 and 10 mg/kg/day, reduced infiltration of inflammatory cells in the central and portal areas, was observed (Figure 3D(.

Histological evaluation by Masson's trichrome staining in LPS group showed morphological changes and liver fibrosis, as indicated by disturbance of the tissue design, extension of fibers, and fibers accumulation indicated by a blue color (Figure 4B). Administration of TQ 2 and 5 mg/kg/day reduced collagen deposition in LPS groups (Figures 4 C and D); however, they were not statistically significant as compared to LPS group (Figure 4F). A significant reduction in liver collagen deposition was observed after treatment with TQ 10 mg/kg compared to LPS group (Figures 4 E and F). 

## Discussion

In the present study, we demonstrated the hepatoprotective effects of TQ on liver fibrosis induced by LPS in rats. We found that treatment with different doses of TQ improved liver fibrosis, liver function tests and oxidative stress status.

Fibrosis is one of the most important signs of hepatic injury in several chronic liver diseases induced by a variety of causes (Bataller and Brenner, 2005 ▶). It is characterized by an abnormal accumulation of extracellular matrix proteins that can result in nodule formation and altered hepatic function (Friedman, 2008 ▶). It may lead to cirrhosis, impairment of liver structure and function, and eventually death (Rockey and Friedman, 2012 ▶). Similar to previous studies, in this study, we found that administration of LPS induces high levels of AST and ALT (Harry et al., 1999 ▶). Our data showed the positive effect of TQ as reflected by a significant reduction in serum AST and ALT concentrations. It has been indicated that elevated Alk-P levels are regarded as an index of liver damage. In fact, this enzyme is a part of the endogenous defense system against LPS by removing phosphate groups from LPS, thus, attenuating the toxicity of this molecule (Koyama et al., 2002 ▶). Therefore, cholestasis, sepsis and other liver injuries like fibrosis are characterized by elevated serum Alk-P levels. Damaged liver fails to excrete alkaline phosphatase made in bone, intestine and liver. Consequently, elevated serum level of liver Alk-P indicates liver injury like fibrosis. In the present study, we found that treatment with TQ especially at the dose of 10 mg/kg decreased serum Alk-P.

To find the effect of TQ on liver fibrosis in LPS-induced inflammation, we evaluated the histological samples stained by H&E as well as Masson's trichrome. We showed that administration of TQ lowered infiltration of inflammatory cells, fibrosis and collagen content, dose-dependently. Oxidative stress is one of the major factors in the etiology of inflammation injury induced by LPS in liver fibrosis (Karaa et al., 2008 ▶). Also, we found higher MDA and lower antioxidant markers including total thiol groups levels, SOD and catalase activity. It has been demonstrated that LPS is capable of generating oxygen radicals, increasing MDA level and impairing antioxidative defense system through affecting TLR expressed on kupffer cells and HSCs in liver (Karaa et al., 2008 ▶). In this research, administration of TQ to LPS group restored oxidative/antioxidative balance demonstrated by reduced MDA level, and increased SOD and catalase activity in liver tissue. Elevation of intracellular concentration of superoxide ion and hydroxyl radical as a result of SOD and catalase inactivation, exert deleterious effects such as loss of cell membrane integrity and membrane function. Decrease in tissue lipid peroxidation following administration of TQ is due to elevated SOD and CAT activities (Alsaif, 2007 ▶). These results are in agreement with those of the previous studies indicating that TQ is an efficient radical scavenger and plays an important role in liver physiology (Darakhshan et al., 2015 ▶). It is demonstrated that TQ improves liver injury in different models such as cholestatic liver diseases (Oguz et al., 2012 ▶), carbon tetrachloride hepatotoxicity (Hassanein and others, 2016 ▶) and nonalcoholic steatohepatitis (NAFLD) (Awad and others, 2016 ▶), through restoring oxidant status, modulation of inflammatory response and increasing liver enzymes activities (Beheshti et al., 2016 ▶). It is suggested that hepatoprotective effects of TQ act through inhibition of production of reactive oxygen species and NF-κB signaling, altering the level of the various eicosanoids and decreasing fibrogenic events (Bai et al., 2013 ▶; Oguz et al., 2012 ▶).

In conclusion, treatment with TQ in an inflammation-induced model, improved liver fibrosis and liver function tests which is possibly due to improvement of antioxidant defenses and reduction in oxidative damage. More studies are needed to clarify the exact effect of TQ on liver in different diseases. 
